# Physical Activity Pattern Among the Adolescents of a Rural Community in West Bengal

**DOI:** 10.4103/0970-0218.58404

**Published:** 2009-10

**Authors:** Sima Roy, Aparajita Dasgupta

**Affiliations:** Department of Community Medicine, IPGME&R, Kolkata, India; 1 Department of Preventive and Social Medicine, All India Institute of Hygiene and Public Health, Kolkata, India. E-mail: mdsroy06@yahoo.com

Sir,

Physical activity is one of the major lifestyle-related health determinants. However, such an important health-protecting behavior is seen to decline during adolescence. This study was undertaken to assess the physical activity pattern among adolescents and to find out the various factors influencing the same.

An observational study was carried out among the adolescents in Diara village, Singur block, Hoogly district, West Bengal. The total adolescent population was nearly 450 out of the total population of 2252, that is approximately 20%. Of them, 25% were studied by selecting through simple random sampling from a suitable sample frame and interviewing with a questionnaire developed from International Physical Activity Questionnaire.(1)

The day's activity was characterized into type of activity and its duration.

Energy expenditure for each of the activities was computed by Basal Metabolic Rate (BMR) for that duration x metabolic constant for that activity

Total energy expenditure was computed by adding the full-day energy expenditure for all the activities with energy expenditure for growth. This value was compared with the standard total daily energy expenditure as per WHO guidelines.(2)

Seventy percentage of the studied population were students (mostly the younger adolescents), 16% were carpenters, shop helpers, or housemaids, and 14% were unemployed(mostly the older girls). Approximately, they spent 10 h for their occupation, 1 h for travelling (cycling or walking), 1 h for household activities, 2 h for leisure, 2 h for miscellaneous activities, and 8 h for sleeping. Their household activities included sweeping, washing, cooking, cattle caring, looking after children and older people. Girls were more engaged in housework. Leisure activities of younger girls and boys were mainly games. Older girls spent most of their leisure time by watching TV, sewing, and gossiping. Seventy-eight percentage were sufficiently active, whereas 22% were less active. Of these, 70% had a normal BMI. None were found to be obese. Younger adolescents and school attendees were more active and the finding was statistically significant. Physical activities and gender did not show any statistically significant relationship [Table 1].

**Table 1:** Relationship between the level of physical activity and various factors (*n* = 100)

Level of physical activity	Age in years			Sex			School attendance		
		
	10 to 14	15 to 19	Total	Male	Female	Total	Present	Absent	Total
Sufficiently active*	40	38	78	39	39	78	60	18	78
Less active†	6	16	22	13	9	22	10	12	22
Total	46	54	100	52	48	100	70	30	100
	χ_2_ = 3.9 *P* <.05			χ_2_ = .43 *P* >.05			χ_2_ = 8.07 *P* <.05		

* Daily energy expenditure were equal to or more than the expected

† Expended less than expected[7]

The study showed that most of the adolescents were sufficiently active, the level being more at younger age, as was also reported by Heath(3) and Kenneth(4) Besides occupational activity, use of active transport, housework, and games helped them to expend energy. Energy expenditure was more among the school attendees and that was probably due to participation in school's physical education program, as was also observed by Gordon-Larsen(5) Although physical activities and gender did not show any statistically significant relationship, leisure activities varied with age and sex. Older girls spent their leisure time for sedentary activities, probably due to lack of parental or social support for games, which was also reported by other studies.(6,7)

The study recommends for parental and social support for physical activities, community-based recreational opportunities, and school attendance for adolescents.
